# *Vibrio cholerae* Pathogenic Clones

**DOI:** 10.3201/eid1111.041170

**Published:** 2005-11

**Authors:** Anna Salim, Ruiting Lan, Peter R. Reeves

**Affiliations:** * University of Sydney, Sydney, New South Wales, Australia; † University of New South Wales, Sydney, New South Wales, Australia

**Keywords:** Vibrio cholerae, US Gulf Coast strains, pandemic clones, evolution, classical, El Tor, toxigenic, Australian strains, prepandemic strains, dispatch

## Abstract

We resolved the relationships between 2 pandemic clones of *Vibrio cholerae*. Using 26 housekeeping genes, we showed that the US Gulf clone, the Australian clone, and 3 El Tor strains isolated before the seventh pandemic were related to the seventh pandemic clone. The sixth pandemic clone was well separated from them.

Cholera caused by *Vibrio cholerae* is a major disease that has caused great fear since the first recorded pandemic in 1817 because of the frequency of death and the rapidity with which it occurs (*1**,**2*). Approximately 200 O antigens have been distinguished serologically (*3**,**4*), but only O1 and O139 have been found in epidemic and pandemic cholera isolates (*5**,**6*).

The seventh pandemic (1961–present) is still widespread and has a severe impact on 3 continents. The sixth pandemic ended in 1923, but the clone persisted at least until the 1990s (*7*). Furthermore, several cholera outbreaks were reported after the sixth pandemic retreated but before the start of the seventh pandemic. Isolates from these outbreaks were recognized as different from those of the sixth pandemic and were allocated to the El Tor biotype, while the sixth and fifth pandemics, both of which had been studied microbiologically, were referred to as the classical biotype. These El Tor outbreaks occurred in Indonesia and the Middle East (1926–1960) (*5*) and are often referred to as prepandemic isolates because they were later seen as forerunners of the seventh pandemic, which was also of the El Tor biotype. However, now that environmental *V. cholerae* has been studied in some detail, major components of the El Tor phenotype are known to be present in most environmental isolates, and the classical biotype is believed to have arisen by loss of characters otherwise widely present in the species (*8*). Also, cases of sporadic indigenous cholera have been detected in Australia (*9*) and the United States (*10*), both of the O1 El Tor biotype. These are generally referred to as the US Gulf and Australian clones. All of the pathogenic forms discussed above had the O1 serotype, but in 1992 a variant of the seventh pandemic appeared with a new O antigen, O139; this variant is known as *V. cholerae* O139 Bengal (*11*).

The relationships of *V. cholerae* have been studied in several ways, but the most useful insights have come from multilocus enzyme electrophoresis (*12*) and more recently by multilocus sequence analyses (*4**,**13**,**14*). In this study, we sequenced 26 housekeeping genes from *V. cholerae* isolates representative of the sixth and seventh pandemic clones and other closely related toxigenic strains to determine relationships to better understand the origins of pandemic clones.

## The Study

Twenty-six housekeeping genes distributed evenly on both chromosomes (Table A1) were studied by using 5 nontoxigenic environmental isolates and 12 toxigenic *V. cholerae* isolates, which comprise 5 sixth and seventh pandemic isolates and 7 pathogenic isolates related to them (Table). One of the 3 seventh pandemic isolates is N16961; the sequence of its genome (*15*) was used in this study. All 26 housekeeping genes were successfully sequenced from the remaining 11 toxigenic isolates. However, 4 genes could not be amplified by polymerase chain reaction from 1 of the environmental isolates (*sdaA* for 370-94, *hmpA* for 905-93, *glgX* for 928-93, and *pepN* for 370-94) and were not sequenced. The GenBank accession numbers for the nucleotide sequences determined in this study are DQ020969–DQ021380.

The 5 nontoxigenic environmental isolates had different sequences for each gene. These sequences were not found in any of the 12 toxigenic isolates. The average pairwise difference among the 5 environmental isolates in the 22 genes sequenced ranged from 0.67% to 5.29%, an indication that *V. cholerae* as a species has a high level of sequence variation. However 11 of the 26 housekeeping genes (*glyA*, *gppA*, *pntA*, *icd*, *purM*, *plsX*, *ndh*, *glgX*, *adk*, *carA*, and *speA*) were identical in all toxigenic *V. cholerae* isolates, confirming that they are closely related.

**Figure 1:**
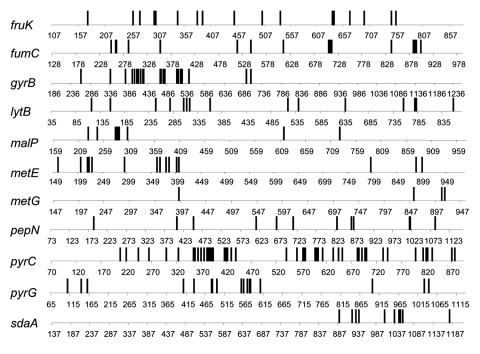
Distribution of base differences in strains of Vibrio cholerae. The base positions relative to the start of the ATG codon are shown. Isolates 395 and E9120 were compared for the fruK, lytB, metE, pepN, pyrG, fumC, malP, metG, pyrC, and sdaA genes, and isolates 395 and E506 were compared for the gyrB gene.

There were 18 mutation/recombination events among the 15 genes with sequence variation in the 12 toxigenic isolates. Three (*malP*, *pyrC*, and *gyrB*) of these genes had undergone 2 changes. Seven cases of single base differences, which are attributed to mutation, were clearly distinct from 11 cases of multiple base differences, which are attributed to recombination. Ten of the events attributed to recombination involve changes in 10 to 51 bases (Figure 1). The *metG* gene, in which the 2 sequences differ by only 4 bases, might have undergone 4 mutational events rather than a single recombination event, but we considered this to be most unlikely.

Some of the 11 genes believed to have undergone recombination may have undergone >1 recombination event. This possibility is likely because 11 of 26 genes have undergone recombination. For example, in *metE* the base changes are at the 2 ends and may represent 2 recombination events; the same applies, to a lesser extent, to *malP*.

With regard to the length of DNA involved in recombination events, segments longer than a gene are most common because only in the genes discussed above and in *sdaA* and *gyrB* are the bases that differed clustered within the gene. This is suggestive of recombination within that gene.

**Figure 2:**
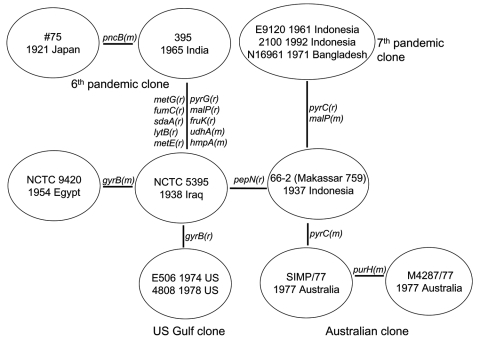
Relationships of toxigenic Vibrio cholerae isolates. The mutational (m) and recombinational (r) changes are given equal weight. Shown is a unique (unrooted) tree, with the events indicated on the branches.

The sequences for the 26 genes were used to produce a tree with mutational and recombinational events and given equal weight, which is shown in Figure 2. By sequencing 26 genes, we have observed sufficient variation to determine the relationships of the isolates studied. The tree is unrooted because the environmental isolates share no alleles with each other or toxigenic isolates. The relatively high rate of recombination in *V. cholerae* means that the level of sequence similarity does not indicate relatedness of the isolates unless the sequences are very similar and differences can be attributed to mutation, as in the toxigenic strains. However, other reasons exist for believing that the root is on the long 10-event branch that includes 2 mutational changes. The 10 El Tor isolates on 1 end are, at most, 3 steps from it and the 2 classical sixth pandemic isolates are separated by 1 event. The isolates date from 1937 to 1992 for the former group and from 1921 to 1965 for the latter group. Since 1 group giving rise to the other while undergoing little divergence itself is highly improbable, we believe that the root lies somewhere on that branch. The properties that characterize the El Tor biotype are those of environmental strains, which makes it unlikely that they are derived from the sixth pandemic, and the reverse seems even less likely because the fifth pandemic was also the classical biotype. We therefore treat the tree as being rooted on the long branch, which enables us to follow the sequence of events.

Among the El Tor isolates, the Australian clone is the most closely related of the current clones to the seventh pandemic clone, with 1 and 2 events along the 2 branches separating them. The Australian clone, although not discovered until 1977, must have arisen before the seventh pandemic and spread to Australia. The prepandemic outbreak isolates are located separately among the surviving El Tor pathogenic clones, with the 1937 Indonesian 66-2 (Makassar 759) isolate located closest to the seventh pandemic clone. The US Gulf clone diverged before the Australian clone and the seventh pandemic clones diverged, with a single recombination event on the branch to the common ancestor of the Australian and the seventh pandemic clones.

The 2 sixth pandemic isolates are well separated from the other strains, differing from them at 10 loci, an average of 5 events per branch. These include recombination events affecting 8 genes. If representative, ≈30% of the genes have undergone recombination during divergence of the sixth pandemic clone and the El Tor group of pathogenic clones that includes the seventh pandemic clone. The extensive divergence between the sixth pandemic and other toxigenic isolates studied indicates a long period since divergence from the common ancestor, which presumably occurred well before the sixth pandemic (1899–1923). In the absence of any intermediates, we cannot allocate individual events to either branch but presume that each is equally likely to have occurred on either branch.

## Conclusions

This study using sequences of 26 genes has resolved the evolutionary relationship of the 2 major pandemic clones of *V. cholerae* and the relationships of the seventh pandemic clone to other pathogenic El Tor clones and isolates. With the relationships established it is clear that study of the prepandemic isolates and Australian clone in particular could illuminate the events involved in the emergence of the current seventh pandemic clone from this lineage.

## Appendix

**Table A1:** Chromosomal location of 26 genes of Vibrio cholerae and primers used*

Gene	Chromosomal location† (kb)	Forward primer	Sequence (5´→3´)	Position‡	Reverse primer	Sequence (5´→3´)	Position§	Amplicon size, bp
*gyrB*	11	4876	TGGTTTTTGAGGTGGTGGAT	116	4877	CGCTTGATTCTTACGGTTAC	1330	1,214
*udhA*	141	4968	GAAAAAGAGAGCAGTGTAGG	106	4969	CGTCCCACTTCATAAGGCAC	1131	1,025
*purH*	280	4970	TCAGCGTATCAGATAAAACC	35	4971	CTTGCCATTCACCACACTCC	1118	1,083
*gppA*	314	4972	GCCGTTTCTTCACCCTTGTA	10	4973	GGATTTTTTGCCAGCCATTC	1416	1,406
*lytB*	732	4974	ATGAGCAAACCAATGAAAAT	1	4975	TTAGGCACTTCAAAGAACAT	936	935
*adk*	1050	4873	CATCATTCTTCTCGGTGCTC	6	4967	CACTCACTTCGCTCACTTGC	620	614
*metG*	1102	4878	CTGAACCCCGAAAAAGATAG	31	4879	CTGTGGAAATAAACGATGTC	1050	1,019
*icd*	1209	4965	TTCATTACTGCCGATTATTC	66	4966	TTTGGTGTCTTTCTGCTTAC	1129	1,063
*pepN*	1602	5054	CACACCTCAAGCCAAATACC	9	5055	GTACAGCTTCATGCCACGTT	1219	1,210
*fumC*	1684	4874	TTATCAAGCACAAACTCAAC	66	4875	TCACAGGCAGCATCACATTA	1079	1,013
*metE*	1835	4987	TTGCTCTTGAAAAATACTGG	59	4988	GAGAAGACTTACGCGCAACA	1169	1,110
*ndh*	2038	4989	TTGAGGATTGTTGTTGTGAC	1	4990	GTTTTAATCATGCCGTGCAG	1242	1,241
*plsX*	2177	5009	ATTTAACCGTTGCACTTGAT	8	5010	GGCTGGGTACTTGTCGTTTC	980	972
*purM*	2380	4991	GTGAGCGGTAATAATCCATC	1	4992	GCTTCGTTGCTATTGGCGGC	1023	1,022
*carA*	2557	4993	CAGATGGTATTTCCGTCGGT	65	4994	AAAGTGGTCAAACAGCGGTG	1111	1,046
*pyrG*	2627	4995	GCGGGGTTGTATCCTCTCTA	29	4996	TATTTGGTGTTTTTATTGAA	1230	1,201
*malP*¶	12	5056	GAAACCTACTCAACAGAAAC	3	5057	CAGAGTGTGGTTGGTGTAAG	1090	1,087
*pncB*¶	105	4999	GTTTTCTCCCCACATCATTC	15	5000	TTCACACATGGCTTTTTCCG	1189	1,174
*hmpA*¶	202	5001	TGCTCACCCAAGAACACATC	2	5002	CGGACCACACAAATAGAA AT	1081	1,079
*glyA*¶	296	5003	ACACCCGCCAAAATGAACAG	110	5004	CATAGGCTTCTCTTCGTCAA	1111	1,001
*fruK*¶	446	5005	TAACGGGCAGTGTAAACCAG	47	5006	GCGGATACTTGAATTTGTTG	930	883
*pntA*¶	500	5049	AGAACAACTCGCTGGTGAAA	21	5050	ATGCCCCTACTACAATGATG	1409	1,388
*sdaA*¶	602	4976	AAGGGGAAAGTTATGCTCTC	49	4977	CATTTGCTGGTGCGCTTGAG	1308	1,259
*speA*¶	761	5058	TAATCCTCTCTGTTTTTGTG	47	5059	TGTTTTTCAGTAGCATTGGC	1214	1,167
*pyrC*¶	878	5013	TGACAACACTGACGATTACA	26	5014	TTGCGAGGTAGGCCGTAGAA	936	910
*glgX*¶	977	5060	GTCAACGCCTTTCCCATTAG	15	5061	TTCGGCAATCAGCTTTACTT	1051	1,036

*The annealing temperatures were 55–62°C for primers for *gyrB* and *pepN* and 55°C for the remaining primers. †Location of each gene on the 2 chromosomes (large and small chromosomes) of *V. cholerae* isolate N16961. ‡Position of the first base of the forward primer relative to the first base of the start codon. §Position of the first base of the reverse primer relative to the first base of the start codon. ¶Genes on the small chromosome of *V. cholerae* isolate N16961.

**Table:** Isolates of Vibrio cholerae tested

Original name	Laboratory name	Clone/isolate	Year isolated	Location	Source*	Serogroup	Biotype
#75	M967	6th pandemic	1921	Japan	CDC	O1	Classical
395	M1616	6th pandemic	1965	India	CVD	O1	Classical
E506	M794	US Gulf Coast	1974	Texas, USA	CVD	O1	El Tor
4808	M796	US Gulf Coast	1978	Louisiana, USA	CVD	O1	El Tor
NCTC 9420	M640	Pre-7th pandemic	1954	Cairo, Egypt	NCTC	O1	El Tor
NCTC 5395	M543	Pre-7th pandemic	1938	Baghdad, Iraq	NICED	O1	El Tor
66-2 (Makassar 759)	M802	Pre-7th pandemic	1937	Sulawesi, Indonesia	IP	O1	El Tor
SIMP/77	M2140	Australian	1977	Australia	QH	O1	El Tor
M4287/77	M2141	Australian	1977	Australia	QH	O1	El Tor
2100	M663	7th pandemic	1992	Bali, Indonesia	IMVS	O1	El Tor
E9120	M793	7th pandemic	1961	Indonesia	CVD	O1	El Tor
N16961†		7th pandemic	1971	Bangladesh	GenBank	O1	El Tor
1085-93	M549	Environmental	1993	Germany	NIHJ	O37	
141-94	M553	Environmental	1994	Germany	NIHJ	O70	
905-93	M555	Environmental	1993	Argentina	NIHJ	O97	
928-93	M557	Environmental	1993	Argentina	NIHJ	O6	
370-94	M563	Environmental	1994	South Korea	NIHJ	O81	

*CDC, Centers for Disease Control and Prevention; CVD, Centre for Vaccine Development (Dr James Kaper); NCTC, National Collection of Type Cultures; NICED, National Institute for Cholera and Enteric Diseases; IP, Institute Pasteur (Dr A. Dodin); QH, Queensland Health (Dr Denise Murphy); IMVS, Institute of Medical and Veterinary Science; NIHJ, National Institute of Health, Japan (Dr Tohio Shimada). †Sequence of the genome of isolate N16961 was used. GenBank accession numbers for chromosomes 1 and 2 are AE003852 and AE003853, respectively.
